# Taurine and tea polyphenols combination ameliorate nonalcoholic steatohepatitis in rats

**DOI:** 10.1186/s12906-017-1961-3

**Published:** 2017-09-08

**Authors:** Wenhua Zhu, Siwen Chen, Ronggui Chen, Zhiqing Peng, Jun Wan, Benyan Wu

**Affiliations:** Gastroenterology Department, NanLou Clinic, General Hospital of People’s Liberation Army, Beijing, 100853 People’s Republic of China

**Keywords:** Nonalcoholic steatohepatitis, Taurine, Tea polyphenols

## Abstract

**Background:**

Nonalcoholic steatohepatitis (NASH) is a progressive form of nonalcoholic fatty liver disease, for which there is currently no safe and effective drug for therapy. In this study, we explored the effects of taurine, tea polyphenols (TPs), or a combination thereof, on NASH rats.

**Methods:**

Rats were divided into a normal group, a high-fat diet induced model group and a treatment group (including taurine, TPs, or taurine + TPs treatment for 8 weeks). Twelve weeks later, all rats were sacrificed, and serum transaminase, lipid and lipopolysaccharide levels and hepatic oxidative stress levels were determined. Histological changes were evaluated.

**Results:**

In NASH rats, hepatocyte damage, lipid disturbance, oxidative stress and elevated lipopolysaccharide levels were confirmed. Taurine treatment alleviated hepatocyte damage and oxidative stress. TPs treatment improved lipid metabolism and increased hepatic antioxidant activity. The therapeutic effects of taurine + TPs treatment on hepatocyte damage, lipid disturbance, and oxidative stress were superior to those of taurine and TPs treatment, respectively. Taurine, TPs and their combination all decreased serum lipopolysaccharide levels in NASH rats, but the combination of the compounds caused these levels to decrease more significantly than taurine or TPs treatment alone.

**Conclusion:**

Taurine combined with TPs treatment could relieve NASH by alleviating hepatocyte damage, decreasing oxidative stress and improving lipid metabolism and gut flora disturbance partly. Taurine and TPs combination may act as a new effective medicine for treating NASH patients.

## Background

Nonalcoholic fatty liver disease (NAFLD) is a clinical pathological syndrome defined as diffuse hepatocyte steatosis and fat storage in the hepatic lobule without excessive alcohol intake [1]. NAFLD includes a spectrum of disorders ranging from simple steatosis, or nonalcoholic fatty liver (NAFL), to its progressive form, known as nonalcoholic steatohepatitis (NASH). NASH is associated with hepatic fibrosis leading to cirrhosis, and its complications include hepatocellular carcinoma. NASH is a chronic progressive liver disease that is characterized pathologically by steatosis combined with nonspecific inflammation, hepatocellular ballooning and mixed inflammatory cell infiltration [1].

The epidemiological surveys from Guangzhou, Shanghai, Hongkong and other developed areas of China showed that the prevalence of NAFLD is approximately 15% [2]. NAFLD is associated with an increased standardized mortality ratio compared to that of the general population and has become the main cause for liver transplantation in the United States [1].

The “two-hit hypothesis” of NAFLD pathogenesis is widely accepted [3]. Hepatocyte steatosis caused by insulin resistance (the first hit) results in insufficient viability in hepatocytes and triglyceride accumulation in the liver, which together provide an optimal microenvironment for further lipid peroxidation/oxidative stress (the second hit), which impairs mitochondrial function, promotes the activation and proliferation of hepatic stellate cells, and results in pathological changes from simple steatosis to hepatic inflammation and fibrosis.

A pharmacological approach is necessary to treat NASH because of failure to cure it using only changes in diet and lifestyle [4]. For NAFLD with hepatic inflammation (mainly NASH), antioxidative drugs should be used to prevent chronic liver disease progression. Currently, betaine, berberine, curcumin, silybin, vitamin E and some other antioxidative drugs that have not been widely used in clinical practice have been used in clinical therapy trials for NASH. These drugs reduced the serum transaminase levels, alleviated steatosis, but there was no sufficient evidence of inflammation alleviation [1]. Therefore, it is urgent that we find a more effective medicine against NAFLD.

Taurine (2-aminoethane sulfur acid), a type of beta amino acid, can be extracted easily from marine shellfish and fish; taurine plays important roles in many essential biological processes, and the liver is one of its target organs. Chen et al. [5] reported that taurine could significantly decrease blood glucose, blood lipid and serum transaminase levels, and alleviate hepatic steatosis in NAFLD patients. The high fat diet-induced NASH rat model has been widely used to observe the efficacy of drugs. In previous studies, we found that taurine could decrease the body and liver weights of NAFL rat models and alleviate steatosis and inflammation [6], which were also proven by Gentile et al. [7]. However, we found that the therapeutic effect of taurine on NASH rats was weaker than that on NAFL rats. A previous study showed that the therapeutic effect of taurine and silybin on NASH patients was more significant than that of taurine and essentiale [8]. These studies show that taurine combined with antioxidative drugs is a more effective treatment that resulted in significant improvement in NASH.

Tea polyphenols (TPs) are recognized as natural antioxidants and are complex compounds extracted from fresh tea. In recent years, many studies have indicated that TPs have good therapeutic effects on fatty liver disease [9]. We undertook preliminary experiments, which showed that TPs treatment (600 mg/Kg/d) could significantly decrease the levels of serum low-density lipoprotein cholesterol (LDL-C) and total cholesterol (TC) in NASH rat models, TPs treatment also caused side effects such as decreased activity, dull hair, and severe weight loss. Unexpectedly, there were no significant pathological improvements in hepatic steatosis after TPs treatment; moreover, TPs treatment aggravated pathological inflammatory infiltration in NASH rats. These results indicate that there are no therapeutic effects of 600 mg/Kg TPs in a NASH rat model. We attribute the poor effects of TPs in NASH rats to the following two reasons: One reason may be the overdose or unsuitable administration of TPs. TPs reduces the serum cholesterol levels mainly through reducing the intestinal absorption of cholesterol, which is important for normal physiological functions of the body. While an overdose or the unsuitable administration of TPs used in the study may result in lower serum cholesterol levels, normal physiological functions may also be affected. Therefore, more appropriate administration and doses of TPs may improve the therapeutic effects of TPs on NASH. This hypothesis is supported by other investigators [10]. The other reason may be the bioavailability of natural TPs. It is recognized that lipid peroxidation in liver cells is a key event in the pathogenesis of NASH, although they are naturally water-soluble compounds, TPs can not penetrate liver cell membranes to reduce reactive oxygen species (ROS), and have subsequent anti-peroxidation effects. Previous research by Lambert et al. [11] showed that water-soluble TPs were poorly bioavailable in humans and rodents, and the transdermal delivery of TPs could improve bioavailability. Inspired by this, we hypothesized that the transformation of water-soluble TPs into a lipid-soluble form could allow TPs to bind easily with cell membranes and directly inhibit intracellular ROS. The aim of this study was to evaluate whether taurine combined with TPs (water-soluble form or lipid-soluble form) treatment had a protective effect on high-fat diet-induced NASH in rats, and whether combination of TPs and taurine would be more effective than either substance alone. Furthermore, we would investigate the possible mechanisms of the effects of taurine, TPs, or a combination thereof, on NASH rats. Research results were expected to provide new therapeutic agents for use in NASH patients.

## Methods

### Preparation of taurine and tea polyphenols (TPs) combination

To prepare taurine and water-soluble TPs (wsTP) combination (Tau + wsTP), appropriate amounts of Tau and wsTP were dissolved in water. The final concentrations of Tau and wsTP was 120 mg/ml and 67 mg/ml, respectively. The steps for Tau and lipid-soluble tea polyphenols (lsTP) combination (Tau + lsTP) preparation were as follows: (1) sodium stearate was dissolved in water (sodium stearate concentration: 20 mg/ml); (2) lsTP was added; (3) the mixture was heated (40 °C) and stirred by a magnetic heating agitator to obtain a lsTP and sodium stearate suspension liquid; (4) Tau was added; and (5) the mixture was heated (40 °C) and stirred until there were no deposits. The final concentrations of Tau and lsTP were 120 mg/ml and 67 mg/ml, respectively.

Tau, wsTP and lsTP were all from the Yuanchenggongchuang Science and Technology Limited Company (Wuhan, Hubei, China).

### Animal experiments

One hundred and twenty specific pathogen-free male Sprague-Dawley rats (215 ± 20.1 g) were obtained from the Center for Laboratory Animal Science of Guangdong Province and were divided randomly into 8 groups. Each group was matched by body weight, the sample size of each group (*n* = 15) was determined by statistical formula: (1) the normal group was fed a standard diet for 12 weeks; (2) the model group was fed a high-fat diet (standard diet +10% lard +2% cholesterol); and (3) the model control group was fed the high-fat diet mentioned above with sodium stearate (0.4 ml for each rat per day) feeding from the 29th day to the end of the 12th week; (4) the Tau treatment group was fed the high-fat diet for 12 weeks, and received the tau solution (120 mg/ml, 125 mg/kg·day) by gavage from the 29th day to the end of the 12th week; (5) the wsTP treatment group was fed the high-fat diet for 12 weeks, and received the wsTP solution (67 mg/ml, 75 mg/kg·day) by gavage from the 29th day to the end of the 12th week; (6) the lsTP treatment group was fed the high-fat diet for 12 weeks, and received the lsTP solution (suspended with sodium stearate) (67 mg/ml, 75 mg/kg·day) by gavage from the 29th day to the end of the 12th week; (7) the Tau + wsTP treatment group was fed the high-fat diet for 12 weeks, and received the Tau + wsTP (Tau 62.5 mg/kg·day and wsTP 37.5 mg/kg·day) combination by gavage from the 29th day to the end of the 12th week; (8) the Tau + lsTP treatment group was fed the high-fat diet for 12 weeks, and received the Tau + lsTP (Tau 62.5 mg/kg·day and lsTP 37.5 mg/kg·day) combination by gavage from the 29th day to the end of the 12th week.

To determine the proper dosage of TPs in rats, previous study was referred to. The main component of TPs is catechins, which account for 80% of TPs. The catechins include epicatechin, epigallocatechin, epicatechin-3-gallate, and epigallocatechin-3-gallate (EGCG). Among them, EGCG accounts for over 60% of the catechins, and maybe the most biologically active component that is responsible for the most of the pharmacologic effects of TPs. Wong et al. [12] found that 50 mg/kg·day EGCG by intraperitoneal injection could modulate immune function in mice and had a low toxicity; high-performance liquid chromatography detection showed that plasma EGCG concentrations peaked between 5 and 30 min, and returned to baseline levels below detection limit by 24 h post-injection, which was comparable to untreated controls. It is well known that the administration dosage in mice is higher than that in rats and that oral administration is widely used in clinical applications, so 75 mg/kg·day TPs by gavage in rats was used in the present study.

All rats were fed the diets ad libitum, given tap water, and kept in metal cages (5 rats per cage) in a room with a controlled temperature (22 ± 1 °C) and humidity (65%–70%), under a 12:12-h lightdark cycle. All animals received humane care in compliance with institutional animal care guidelines. This experiment was approved by the medical ethics committee in General Hospital of People’s Liberation Army (No. 2015HN16). At the end of 12 weeks, all rats were weighed and sacrificed via overdose of chloral hydrate injected into the abdominal cavity after overnight fasting. Blood samples were collected by cardiac puncture. Intact livers were taken out of the abdominal cavity for weighing and macropathological observation and then lavaged with ice-cold 0.9% saline. After that, the right lobes of the livers were dissected and some pieces were soaked in 10% neutral buffered formalin for histological observation.

### Serum biochemical analysis

Serum transaminase (alanine aminotransferase [ALT] and aspartate aminotransferase [AST]) activities and total cholesterol (TC), triglyceride (TG) and low-density lipoprotein cholesterol (LDL-C) levels were detected with kits from the Jiancheng Bioengineering Institute (Nanjing, China). Serum endotoxin/lipopolysaccharide (LPS) were detected with kits from Sigma (Sigma, St. Louis, MO, USA).

### Determinations of oxidative stress levels

Liver pieces were homogenized in ice-cold 0.15 M KCl,10% (*w*/*v*). The degree of lipid peroxidation in the liver was assessed by measuring malondialdehyde (MDA) levels using the thiobarbituric acid (TBA) method and superoxide dismutase (SOD) activities using the xanthine oxidase method according to the manufacturer’s instructions. The assay kits for determining MDA levels and SOD activities were from the Jiancheng Bioengineering Institute (Nanjing, Jiangsu, China). Protein levels were determined by Coomassie brilliant blue staining, and bovine serum albumin was used as a standard.

### Histological assessment

The liver samples were fixed in 10% formalin, then sliced into 4- to 6-mm pieces, dehydrated in ethanol, embedded in paraffinwax, sectioned (5-μm thick), and stained with hematoxylin and eosin (HE), oil red, Masson stains. Each section was then separately scored for steatosis and inflammation separately according to the published criteria [6], as shown in Table 1.

**Table 1:** Grade of NASH

Steatosis	Grade 1	Up to 25% steatosis
	2	26% to 50% steatosis
	3	51% to 75% steatosis
	4	More than 76% steatosis
Inflammation	Grade 1	Focal collection of mononuclear cells
	2	Diffuse infiltrates of mononuclear cells
	3	Focal collection of polymorphonuclear cells
	4	Diffuse infiltrates of polymorphonuclear

### Statistical analysis

Results are expressed as the mean ± the standard deviation. The ANOVA and Student’s t-test were used for comparison among the groups. In the analyses of histological grading, nonparametric tests (Wilcoxon test, Kruskal-Wallis H-test and Nemenyi test) were used.

## Results

### Taurine and TPs combination reduces the liver index in NASH rats but not body weights

Ninety-seven rats completed the 12-week experiment, and there were no significant differences in the mortality rats between the groups (*P* > 0.05). There were no significant differences in the initial body weights of the groups (*P* > 0.05). At the end of the 84th day, there were still no significant differences in the body weights of the groups (*P* > 0.05). The results showed that the liver index (liver weight/body weight) increased significantly in the model group compared with that of the normal group or the treatment groups (*P* < 0.01, Table 2), but there were no significant differences between the treatment groups (*P* > 0.05).

**Table 2:** Effect of taurine and/or TPs on liver index in NASH rats

	Nor	Mod	Mod cont	Tau	wsTP	lsTP	Tau + wsTP	Tau + lsTP
Liver index (Mean ± SD)	0.0269 ± 0.0008	0.0331 ± 0.0011^a^	0.0335 ± 0.0014^a^	0.0285 ± 0.0010^b^	0.0291 ± 0.0012^b^	0.0302 ± 0.0007	0.0290 ± 0.0011^b^	0.0288 ± 0.0015^c^
n	13	12	12	12	12	12	12	12

*Nor* normal group, *Mod* model group, *Mod cont* model control group, *Tau* taurine treatment group, *wsTP* water soluble tea polyphenols treatment group, *lsTP* lipid soluble tea polyphenols treatment group, *Tau + wsTP* taurine and water soluble tea polyphenols treatment group, *Tau + lsTP* taurine and lipid soluble tea polyphenols treatment group, *n* number of surviving rats

^a^ *p* < 0.01, compared with Nor; ^b^ *p* < 0.01, compared with Mod; ^c^ *p* < 0.01, compared with Mod cont

### Taurine and TPs combination improves serum biochemical parameters

**Fig. 1:**
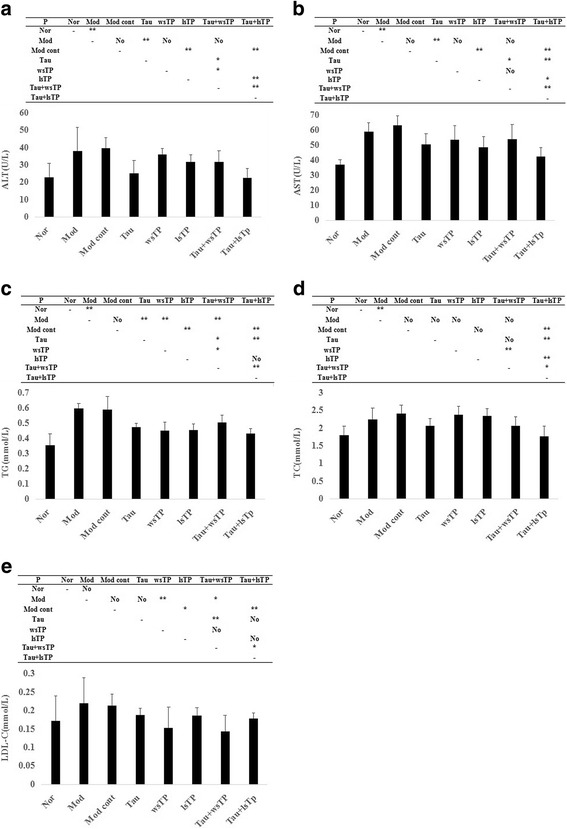
Effect of taurine and/or TPs on serum biochemical parameters of NASH rats. Serum levels of ALT, AST, TC and TG in the model group elevated significantly compared with those in the normal group, but no significant differences of them all were shown between model group and model control group (**a**–**e**). When compared with the model group, taurine treatment decreased serum ALT, AST and TG levels significantly (*P* < 0.05). The serum TG and LDL-C levels decreased significantly in the wsTP treatment group compared with the model group (*P* < 0.05), while there were no significant differences of ALT, AST and TC between the two groups (*P* > 0.05). When lsTP was given to the NASH rats, results showed that serum TG, LDL-C, ALT and AST levels were all decreased significantly (*P* < 0.05 or *P* < 0.01). Furthermore, when taurine and wsTP were given simultaneously to NASH rats, serum TG and LDL-C levels decreased significantly (*P* < 0.05 or *P* < 0.01), the downtrend of TG in the Tau + wsTP group were more significant than that in the taurine treatment group or the wsTP treatment group (*P* < 0.05), and the downtrend of LDL-C in the Tau + wsTP treatment group were more significant than that in the taurine treatment group (*P* < 0.01). When taurine and lsTP were both given to NASH rats, serum ALT, AST, TC, TG and LDL-C levels all decreased significantly (*P* < 0.01), moreover, the downtrend of AST and TC in the Tau + lsTP treatment group were more significant than that in the taurine treatment group or the lsTP treatment group (*P* < 0.05 or *P* < 0.01), the downtrend of ALT in the Tau + lsTP group were more significant than that in the lsTP treatment group (*P* < 0.01), and the downtrend of TG in the Tau + lsTP group were more significant than that in the taurine treatment group (*P* < 0.01). NASH, nonalcoholic steatohepatitis; ALT, alanine aminotransferase; AST, aspartate aminotransferase; TC, total cholesterol; TG, triglyceride; LDL-C, low-density lipoprotein cholesterol; Nor, normal group; Mod, model group; Mod cont, model control group; Tau, taurine treatment group; wsTP, water soluble tea polyphenols treatment group; lsTP, lipid soluble tea polyphenols treatment group; Tau + wsTP, taurine and water soluble tea polyphenols treatment group; Tau + lsTP, taurine and lipid soluble tea polyphenols treatment group; “*” *p* < 0.05; “**” *p* < 0.01; “No” *p* > 0.05

Serum levels of ALT, AST, TC and TG in the model group increased significantly compared with those in the normal group (*P* < 0.05), but no significant differences between these variables were shown between the model group and the model control group (*P* > 0.05). When compared with the model group, taurine treatment decreased serum ALT, AST and TG levels significantly (*P* < 0.05). These results indicate that taurine could alleviate hepatocyte injury and decrease serum lipids in NASH rats. When we focused on the wsTP group, the results showed that serum TG and LDL-C levels decreased significantly in the wsTP treatment group compared with those of the model group (*P* < 0.05), but there were no significant differences in ALT, AST and TC between the two groups (*P* > 0.05). The results illustrated that wsTP could decrease serum TG levels in NASH rats, but could not ameliorate hepatocyte injury. When the NASH rats were treated with lsTP, results showed that not only serum TG and LDL-C levels, but also serum ALT and AST levels decreased significantly (*P* < 0.05 or *P* < 0.01). The results demonstrated that lsTP could ameliorate hepatocyte injury and improve lipid metabolism in NASH rats, and that the therapeutic effect of lsTP on NASH was superior to that of wsTP. Furthermore, when taurine and wsTP were given simultaneously to NASH rats, results showed that serum TG and LDL-C levels decreased significantly (*P* < 0.05 or *P* < 0.01), the downtrend in TG levels in the Tau + wsTP group was more significant than that in the taurine treatment group or the wsTP treatment group (*P* < 0.05), and the downtrend in LDL-C in the Tau + wsTP treatment group was more significant than that in the taurine treatment group (*P* < 0.01). When taurine and lsTP were both given to NASH rats, results showed that serum ALT, AST, TC, TG and LDL-C levels all decreased significantly (*P* < 0.01), moreover, the downtrend in AST and TC in the Tau + lsTP treatment group was more significant than that in the taurine treatment group or the lsTP treatment group (*P* < 0.05 or *P* < 0.01), the downtrend in ALT in the Tau + lsTP group was more significant than that in the lsTP treatment group (*P* < 0.01), and the downtrend in TG in the Tau + lsTP group was more significant than that in the taurine treatment group (*P* < 0.01). These results indicated that taurine could ameliorate hepatocyte injury and improve serum lipid metabolism in NASH rats; the effects mentioned above could be enhanced by TPs (wsTP or lsTP) supplementation, especially with the lsTP supplement (Fig. 1a–e).

### Effects of taurine and TPs combination on hepatic oxidative stress

**Fig. 2:**
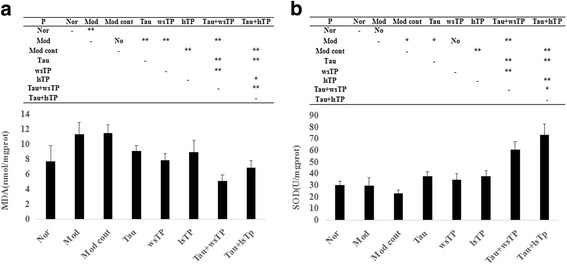
Effect of taurine and/or TPs on hepatic oxidative stress. Hepatic MDA levels in the model group and the model control group increased significantly compared with the normal group (*P* < 0.01), but no significant differences in hepatic SOD activities between each two groups were shown (*P* > 0.05) (**a**, **b**). Taurine treatment could decrease hepatic MDA levels (*P* < 0.01) and increase hepatic SOD activities in NASH rats (*P* < 0.05). After being treated with wsTP, no significant difference in SOD activity was shown compared with the model group (*P* > 0.05), while MDA levels decreased significantly compared with the model group (*P* < 0.01). When lsTP was given to NASH rats, hepatic MDA levels decreased significantly (*P* < 0.01) and hepatic SOD activity increased significantly (*P* < 0.01). To enhance the therapeutic effect of taurine or TPs on NASH alone, taurine and TPs (wsTP or lsTP) combination were used. Results showed that hepatic MDA levels in Tau + wsTP treated and Tau + lsTP treated NASH rats decreased significantly compared with NASH rats (*P* < 0.01), and hepatic SOD activity increased significantly (*P* < 0.01) in both the two group compared with NASH rats. Furthermore, when we compared taurine and TPs (wsTP or lsTP) combination treatment NASH rats with taurine or TPs treatment NASH rats, hepatic MDA levels in the combination treatment rats were lower than those in the taurine or TPs treatment rats (*P* < 0.01 or *P* < 0.05), and hepatic SOD activity in the combination treatment rats were more than those in the taurine or TPs treatment rats (*P* < 0.01). Results illustrated that the therapeutic effects of Tau + wsTP or Tau + lsTP treatment on NASH rats were superior to that of taurine or TPs (wsTP or lsTP) treatment alone. MDA, malondialdehyde; SOD, superoxide dismutase; NASH, nonalcoholic steatohepatitis; Nor, normal group; Mod, model group; Mod cont, model control group; Tau, taurine treatment group; wsTP, water soluble tea polyphenols treatment group; lsTP, lipid soluble tea polyphenols treatment group; Tau + wsTP, taurine and water soluble tea polyphenols treatment group; Tau + lsTP, taurine and lipid soluble tea polyphenols treatment group; “*” *p* < 0.05; “**” *p* < 0.01; “No” *p* > 0.05

Methane dicarboxylic aldehyde (MDA) is a decomposition product of the polyunsaturated fatty acid hydroperoxides that are generated from reactions with ROS [13]. Hence, the MDA level is considered an indirect index of the extent of lipid peroxidation. SOD is an important antioxidant enzyme in nearly all living cells exposed to oxygen. Therefore, SOD activity is considered an indirect index of the extent of protective antioxidant abilities. Hepatic MDA levels in the model group and the model control group increased significantly compared with those in the normal group (*P* < 0.01), but no significant differences in hepatic SOD levels between the two groups were shown (*P* > 0.05). Taurine treatment could decrease hepatic MDA levels (*P* < 0.01) and increase the hepatic SOD activity in NASH rats (*P* < 0.05). After treatment with wsTP, no significant difference in SOD activity was shown compared with the model group (*P* > 0.05), while MDA levels decreased significantly compared with those in the model group (*P* < 0.01). When lsTP were given to NASH rats, hepatic MDA levels decreased significantly (*P* < 0.01) and hepatic SOD activity increased significantly (*P* < 0.01). These results indicated that the high-fat diet promoted lipid peroxidation injury and impaired antioxidant activity in the rat livers, and both of these effects in NASH rats were improved by taurine or lsTP treatment; however, lipid peroxidation injury in NASH rats was ameliorated by only wsTP treatment. To enhance the therapeutic effects of taurine or TPs alone on NASH, taurine and TPs (wsTP or lsTP) compounds were used in combination. The results showed that hepatic MDA levels not only in Tau + wsTP treated NASH rats but also in Tau + lsTP treated NASH rats, decreased significantly compared with those in NASH rats (*P* < 0.01), and hepatic SOD activity increased significantly (*P* < 0.01) in both groups compared with that in NASH rats. Furthermore, when the hepatic MDA levels in the taurine and TPs (wsTP or lsTP) combination treated NASH rats were compared with those in the taurine or TPs treated NASH rats, data showed that hepatic MDA levels in the combination treated rats were less than those in the taurine or TPs treated rats (*P* < 0.01 or *P* < 0.05), and the hepatic SOD activity in the combination treated rats were greater than that in the taurine or TPs treated rats (*P* < 0.01). These results illustrated that the therapeutic effects of Tau + wsTP or Tau + lsTP treatment on NASH rats were superior to those of taurine or TPs (wsTP or lsTP) treatment alone (Fig. 2a and b).

### Taurine and TPs combination diminished LPS level in NASH rats

**Fig. 3:**
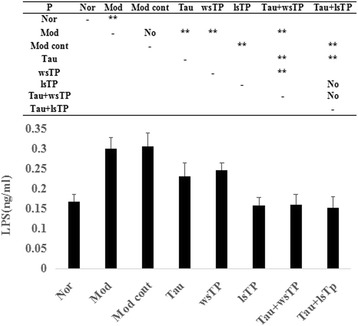
Effect of taurine and/or TPs on serum LPS level in NASH rats. Results illustrated that high-fat diet increased serum LPS levels significantly compared with the normal group (*P* < 0.01). When treated NASH rats with taurine, TPs (wsTP or lsTP), or taurine and TPs (wsTP or lsTP) combination, serum LPS levels all decreased significantly (*P* < 0.01), moreover, the LPS levels in NASH rats treated with taurine and wsTP combination were lower than those in NASH rats treated with taurine or wsTP alone (*P* < 0.01). The results mentioned above implied that both taurine and TPs (wsTP or lsTP) could decrease serum LPS level in NASH rats, and the combination of taurine and wsTP showed more superior therapeutic effect on NASH than taurine or wsTP alone. LPS, lipopolysaccharide; NASH, nonalcoholic steatohepatitis; Nor, normal group; Mod, model group; Mod cont, model control group; Tau, taurine treatment group; wsTP, water soluble tea polyphenols treatment group; lsTP, lipid soluble tea polyphenols treatment group; Tau + wsTP, taurine and water soluble tea polyphenols treatment group; Tau + lsTP, taurine and lipid soluble tea polyphenols treatment group; “**” *p* < 0.01; “No” *p* > 0.05

To date, several research groups have identified that LPS plays a role in the development of NASH [14], so serum LPS levels were detected in this study. The results illustrated that the high-fat diet increased serum LPS levels significantly compared with those of the normal group (*P* < 0.01). When NASH rats were treated with taurine, TPs (wsTP or lsTP), or taurine and TPs (wsTP or lsTP) combination, serum LPS levels all decreased significantly (*P* < 0.01); moreover, the LPS levels in NASH rats treated with taurine and wsTP combination were lower than those in NASH rats treated with taurine or wsTP alone (*P* < 0.01). The results mentioned above implied that both taurine and TPs (wsTP or lsTP) could diminish serum LPS levels in NASH rats, and the combination of taurine and wsTP had a superior therapeutic effect on NASH compared to taurine or wsTP alone (Fig. 3).

### Taurine and TPs combination alleviated steatosis and inflammation in the NASH liver

**Fig. 4:**
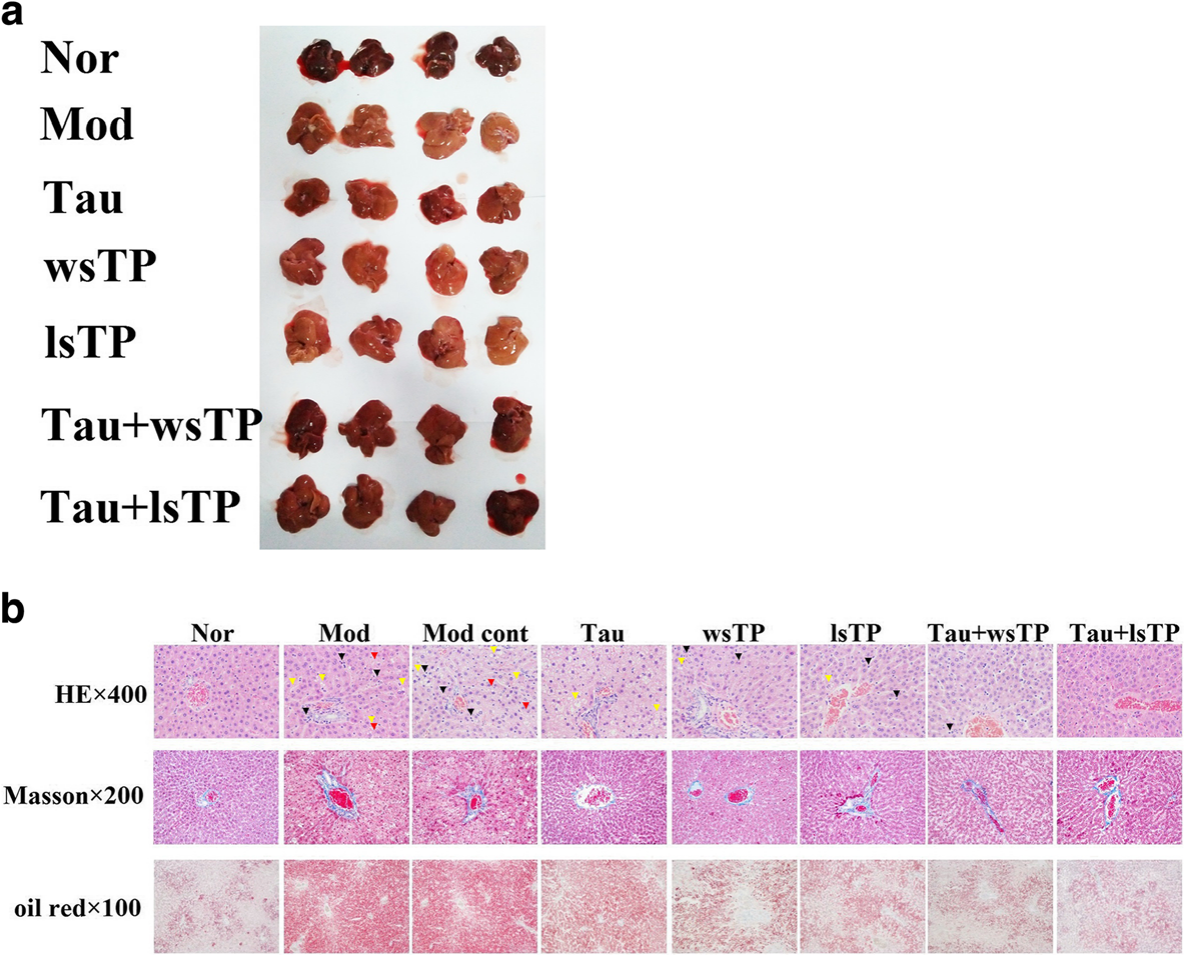
Effect of taurine and/or TPs on histopathological changes in NASH rats. Gross pathological observation showed ruddy liver in the normal group, and yellow hepatomegaly in the model group and model control group. When treated NASH rats with taurine, wsTP, lsTP, taurine + wsTP, or taurine + lsTP, color of liver turned red more or less. When compared with taurine treatment group, wsTP treatment group or lsTP treatment group, respectively, the color of liver in the combination treatment groups (taurine + wsTP and taurine + lsTP) appeared redder (**a**). In the taurine treatment group, liver histological examinations showed normal liver or mild microvesicular steatosis without inflammatory infiltration or fibrosis in the normal group. Severe macro- and microvesicular steatosis (yellow arrow), infiltration of mild lobular mixed neutrophilic and mononuclear cells (black arrow), even focal necrosis, perisinusoidal and portal fibrosis, severe hepatocellular ballooning (red arrow) were observed in the model group and model control group. There were remarkable histological improvements in steatosis and inflammation in taurine treatment group, wsTP treatment group, lsTP treatment group, taurine + wsTP combination treatment group and taurine + lsTP combination treatment groups (*P* < 0.05), compared with model group or model control group. In addition, the histological improvements in combination treatment group were more remarkable than in taurine group, wsTP group and lsTP group, respectively (*p* < 0.05) (**b**). NASH, nonalcoholic steatohepatitis; Nor, normal group; Mod, model group; Mod cont, model control group; Tau, taurine treatment group; wsTP, water soluble tea polyphenols treatment group; lsTP, lipid soluble tea polyphenols treatment group; Tau + wsTP, taurine and water soluble tea polyphenols treatment group; Tau + lsTP, taurine and lipid soluble tea polyphenols treatment group; HE, hematoxylin and eosin staining

Macropathological observation revealed ruddy-colored livers in the normal group, and yellow hepatomegaly in the model group and the model control group. When NASH rats were treated with taurine, wsTP, lsTP, taurine + wsTP, or taurine + lsTP, the color of liver turned red, more or less. When compared with those of the taurine treatment group, wsTP treatment group or lsTP treatment group, respectively, the color of the livers in the combination treatment groups (taurine + wsTP and taurine + lsTP) appeared redder (Fig. 4a). Histological examinations showed normal liver or mild microvesicular steatosis without inflammatory infiltration or fibrosis in the normal group. Severe macro- and microvesicular steatosis, infiltration of mild lobular mixed neutrophilic and mononuclear cells, even focal necrosis were observed in the model group and model control group. There were remarkable histological improvements in steatosis and/or inflammation in taurine treatment group, lsTP treatment group, taurine + wsTP combination treatment group and taurine + lsTP combination treatment groups (*P* < 0.05 or *p* < 0.01), except in wsTP treatment group (*P* > 0.05), compared with the model control group (Table 3). In addition, the histological improvements in the combination treatment group were more remarkable than those in the taurine group, wsTP group and lsTP group respectively (*p* < 0.05 or *p* < 0.01) (Table 4) (Fig. 4b).

**Table 3:** Effect of taurine or TPs or their combination on histopathology

	Group	1	2	3	4	χ 2		Group	1	2	3	4	χ 2
Steatosis	Nor	13	0	0	0		Inflammation	Nor	13	0	0	0	
	Mod	0	0	3	9	49.48^a^		Mod	0	0	0	12	53.42^a^
	Mod cont	0	0	1	11	0.11		Mod cont	0	0	0	12	0.00
	Tau	0	6	6	0	14.73^c^		Tau	0	4	8	0	15.06^c^
	wsTP	0	6	6	0	10.73		wsTP	0	4	8	0	9.06
	lsTP	0	8	4	0	14.17^c^		lsTP	0	10	2	0	21.55^b^
	Tau + wsTP	0	9	3	0	16.08^c^		Tau + wsTP	0	8	4	0	15.39^c^
	Tau + lsTP	0	12	0	0	22.50^b^		Tau + lsTP	0	12	0	0	23.38^b^

*Nor* normal group, *Mod* model group, *Mod cont* model control group, *Tau* taurine treatment group, *wsTP* water soluble tea polyphenols treatment group, *lsTP* lipid soluble tea polyphenols treatment group, *Tau + wsTP* taurine and water soluble tea polyphenols treatment group, *Tau + lsTP* taurine and lipid soluble tea polyphenols treatment group. Kruskal–Wallis H-test and Nemenyi test were used in the statistical analysis. ^a^ *P* < 0.01 compared with Normal group; ^b^ *P* < 0.01 compared with Model control group; ^c^ *P* < 0.05 compared with Model control group

**Table 4:** Effect of combination or single treatment on histopathology (u value)

		Tau treatment	wsTP treatment	lsTP treatment	Tau + wsTP treatment
Steatosis	Tau + wsTP treatment	14.54^a^	14.54^a^	/	/
	Tau + lsTP treatment	2.77^a^	/	2.14^b^	1.81
Inflammation	Tau + wsTP treatment	1.60	1.60	/	/
	Tau + lsTP treatment	3.19^a^	/	1.44	2.14^b^

Wilcoxon test was used in the statistical analysis. ^a^ *P* < 0.01; ^b^ *P* < 0.05

## Discussion

NAFLD is a leading cause of chronic liver disease worldwide, especially due to its close relationship with metabolic features, such as type 2 diabetes mellitus and dyslipidemia. In addition to changes in dietary habits and lifestyle, a pharmacological approach is often necessary to treat NASH. So far, few chemical compounds (such as glitazones and vitamin E) have been developed for NASH treatment; however, adverse effects limit their application [15–18]. Taurine and TPs are molecules extracted from natural substances; their combination may help to enhance their separate therapeutic effects on NASH as well as reduce their separate adverse effects. Previous studies have confirmed that taurine has a therapeutic effect on NASH rats, but its effects on NASH are weaker than its effects on simple hepatic steatosis.

In this study, we proved that taurine (125 mg/kg·day), wsTP (75 mg/kg·day), lsTP (75 mg/kg·day), taurine (62.5 mg/kg·day) + wsTP (37.5 mg/kg·day), and taurine (62.5 mg/kg·day) + lsTP (37.5 mg/kg·day) can be used for the prevention and treatment of NASH without side effects (for example, decreased activity and significant weight loss). The results showed that the liver index, and serum levels of TC, LDL-C, ALT and AST in the treatment groups were significantly lower than those in the corresponding NASH rats (*P* < 0.05 or *P* < 0.01). When comparing each combination treatment group with taurine, wsTP or lsTP treatment, Tch, LDL-Ch, ALT and AST levels were decreased significantly (*P* < 0.05 or *P* < 0.01). Furthermore, the therapeutic effects of taurine and lsTP combination treatment on both hepatocytes injury and lipid disturbance were superior to those of taurine and wsTP combination treatment group (*P* < 0.05 or *P* < 0.01).

High-fat diet-induced fatty liver is associated with oxidative stress, which cause lipid peroxidation in hepatocytes. MDA is one of the end-products of lipid peroxidation and causes cytotoxicity, and SOD is one of the antioxidant enzymes. In this study, we observed the effects of taurine, TPs and their combination on the levels of MDA and SOD in NASH rats liver. The results showed that the levels of MDA in the model group and the model control group increased significantly compared to those in the normal group, while the SOD activity remained unchanged. These results prove that lipid peroxidation plays an important role in the progression of NASH. When compared with those in the corresponding control group, the MDA levels in taurine, wsTP, lsTP, taurine + wsTP and taurine + lsTP treatment groups decreased significantly, and the SOD activities increased significantly. In addition, the combination treatment groups showed greater significant differences compared to the single treatment groups. Histological examinations also proved that hepatic fat deposition and inflammatory cell infiltration were alleviated in all the five treatment groups, but especially in the combination groups. The results demonstrated that taurine and TPs can alleviate NASH by improving hepatic oxidative stress.

In addition to the “two-hit hypothesis” foe the pathogenesis of NASH, investigators have proposed the “multiple parallel hits hypothesis” for the pathogenesis of NASH. Inflammation may either precede or follow simple steatosis with multiple factors, many parallel hits may be derived from the gut (mainly gut microbiota) and/or the adipose tissue may promote hepatic inflammation injury [14].

The growing evidence has shown that gut flora disturbance participated in the development of NAFLD, and the recovery of gut flora balance may become a new strategy for the prevention and treatment of NAFLD [19–24]. Endotoxin is part of the Gram-negative bacterial cell membrane, and lipopolysaccharide (LPS) is the active component of endotoxin. Previous studies showed that endotoxin levels were increased significantly in NAFLD patients, which might promote insulin resistance and inflammatory reactions, and LPS supplementation increased serum TG levels significantly and induced hepatocyte steatosis in rats [25, 26]. Wan et al. [27] reported that green tea EGCG could improves epithelial barrier function in monolayers of porcine intestinal epithelial IPEC-J2 cells. In addition, green tea polyphenol had also been confirmed to ameliorate chemotherapeutic agent irinotecan-induced small intestinal mucosa damage in mice [28] and gut ischemia/reperfusion injury in rats [29]. Interestingly, taurine has also been proven to ameliorate intestinal ischemia-reperfusion injury in rats [30] and the chemotherapeutic agent 5-flourouracil induced intestinal mucositis in rats [31].

In this study, we found that LPS levels were increased significantly in NASH model groups compared to those in the normal group and decreased significantly in the treatment groups compared to those in the control groups. What is more, the LPS decreasing effect of the combination treatment is more obvious than that in the single treatment groups. These results suggest that the therapeutic effect of taurine and TPs in NASH rats, at least in part, was attributed to the improvement of gut flora disturbance.

## Conclusion

In summary, we successfully prepared the taurine and TPs combination successfully, and proved that taurine combined with TPs treatment can ameliorate serum biochemical parameters, and histologically improve experimental NASH. The therapeutic effects of taurine combined with TPs in NASH were superior to those of taurine or TPs alone. Further measurements suggested that the improvement of multiple factors participated in the pathogenesis of NASH; in fact, improvement of oxidative stress and gut flora disturbance may account for the therapeutic effects of taurine, TPs and taurine combined with TPs. Further studies must focus on improving the preparation method of taurine and TPs compounds, and their therapeutic and adverse effects on NASH patients must be determined. We propose that taurine and TPs compounds can be a new, effective treatment for NASH patients.

## Abbreviations

ALT: Alanine aminotransferase
AST: Aspartate aminotransferase
EGCG: Epigallocatechin-3-gallate
LDL-C: Low density lipoprotein cholesterol
LPS: Lipopolysaccharide
lsTP: lipid soluble tea polyphenols
MDA: Malondialdehyde
NAFL: Nonalcoholic fatty liver
NAFLD: Nonalcoholic fatty liver disease
NASH: Nonalcoholic steatohepatitis
ROS: Reactive oxygen species
SOD: Superoxide dismutase
TC: Total cholesterol
TG: Triglyceride
TPs: Tea polyphenols
wsTP: water soluble tea polyphenols
